# The Effectiveness of Conventional and Advanced Aligning Archwires: The Insights of Two Randomized Clinical Trials

**DOI:** 10.1055/s-0044-1795080

**Published:** 2025-04-04

**Authors:** Reyam M. Noori, Omar K. Ahmed, Ammar S. Kadhum, Yassir A. Yassir, Marco Di Blasio, Diana Russo, Marco Cicciù, Giuseppe Minervini

**Affiliations:** 1 Department of Orthodontics, College of Dentistry, University of Baghdad, Baghdad, Iraq; 2 Baghdad Al-Karkh Health Directorate, Ministry of Health, Baghdad, Iraq; 3 Department of Orthodontics, School of Dentistry, University of Dundee, Dundee, United Kingdom; 4 Department of Biomedical, Surgical and Dental Sciences, Fondazione IRCCS Cà Granda-Ospedale Maggiore Policlinico, University of Milan, Milan, Italy; 5 Department of Multidisciplinary Medical-Surgical and Odontostomatological Specialties, Oral Surgery Unit, University of Campania “Luigi Vanvitelli”, Naples, Italy; 6 Department of Biomedical and Surgical and Biomedical Sciences, Catania University, Catania, Italy; 7 Saveetha Dental College and Hospitals, Saveetha Institute of Medical and Technical Sciences (SIMATS), Saveetha University, Chennai, India; 8 Multidisciplinary Department of Medical-Surgical and Odontostomatological Specialties, University of Campania “Luigi Vanvitelli,” Naples, Italy

**Keywords:** dentistry, orthodontics, archwires, alignment

## Abstract

**Objectives:**

This study aimed to compare the clinical effectiveness of four aligning archwires: Superelastic Nickel-Titanium (Superelastic-NiTi), SmartArch, Copper-Nickel-Titanium (Cu-NiTi), and Speed Tubular coaxial-Nickel-Titanium (Tubular coaxial-NiTi), regarding the alignment efficiency, associated perception of pain, and possibility of inducing root resorption.

**Materials and Methods:**

This study includes two randomized clinical trials run in parallel. Patients with 5 to 9 mm of mandibular anterior teeth crowding according to Little's irregularity index (LII) who needed fixed orthodontic appliances without extraction were randomly assigned to four groups of aligning archwires (each trial with two groups): 0.014-inch, 0.018-inch Superelastic-NiTi; 0.016-inch SmartArch; 0.014-inch, 0.018-inch Cu-NiTi; and 0.016-inch, 0.018-inch Tubular coaxial-NiTi. LII was measured pretreatment and every 4 weeks for the next 16 weeks. Pain perception was assessed using a visual analogue scale in the first 7 days after the placement of each archwire. Periapical radiographs for mandibular central incisors were taken pretreatment and after 16 weeks to assess root resorption. The alignment efficiency was tested using a repeated measures analysis of variance test with mixed factorial design (between and within-subject effect), while pain perception and root resorption were tested using the Kruskal–Wallis test and Wilcoxon signed-rank test.

**Results:**

A total of 73 patients were recruited from different centers. The analysis included 64 patients who completed the trials. Neither clinical nor statistical significance was found between the groups regarding the alignment. Similarly, there was no significant difference between the four groups regarding pain perception and root resorption. However, root resorption was slightly more in the Superelastic-NiTi group than in the other groups.

**Limitations:**

The short time that root resorption was reported.

**Conclusion:**

The four types of archwires performed comparably regarding the alignment efficiency, associated perception of pain, and the possibility of inducing root resorption.

**Registration:**

The trials included in this study were registered with ClinicalTrials.gov on May 26, 2022 (Registration number: NCT05391542) and August 18, 2022 (Registration number: NCT05510206).

## Introduction

The initial phase of orthodontic treatment involves using the first set of archwires, which effectively align teeth by addressing issues such as crowding and rotation. 1 These archwires, which are known for their remarkable flexibility and superelasticity, are fully inserted into the brackets. 1 2 3 It is necessary for these archwires to exert a light continuous force over an extended duration, and to ensure clinical efficiency. This force must generate optimal tooth movement while causing minimal pain and root resorption. 4 5

The exceptional properties of Nickel-Titanium (NiTi) archwires, including their remarkable springiness, strength, and extensive range of action, have rendered them an ideal choice for the initial phases of orthodontic treatment. 6 To enhance the clinical benefits of NiTi in orthodontics, various components have been incorporated. One among these additions is copper, which serves to reduce the loading stress and, at the same time, offers a relatively elevated unloading stress. This, in turn, may lead to more effective orthodontic movement of teeth. 4 7 Another attempt to enhance the flexibility and decrease the rate of load deflection was to use multistrand archwires, which have been effectively used with stainless steel wires. 8 9 The same concept was used with superelastic NiTi archwires; consequently, a Supercable coaxial superelastic NiTi archwire was introduced. Laboratory tests have demonstrated that these wires exert a force ranging from 36 to 70% of that exerted by solid NiTi archwires. 10 A novel addition to the market was the introduction of Speed six-stranded coaxial tubular superelastic NiTi. 11 The design of the archwire has been enhanced to provide greater flexibility by incorporating a “hollow center” structure. This unique feature enables the archwire to fold over on itself, effectively addressing cases of severe malalignment 12 while exerting only a fraction of the force levels exerted by traditional initial archwires. 10 13

The aforementioned NiTi archwires are characterized by uniformity of composition along the wire. Consequently, these archwires uniformly apply consistent force per deflection to all teeth. 14 To address the issue of uniform force, a solution was sought through the implementation of patented pulsating laser technology. This innovative approach results in developing SmartArch wires featuring distinct force zones. 15 According to the manufacturer's claims, SmartArch utilizes optimal forces to reposition teeth effectively and consequently decreases the duration of treatment due to its incorporation of seven preprogrammed zones designed to apply appropriate forces to individual teeth. 16 17

Various clinical trials have investigated the efficiency of archwires. 4 7 18 19 20 The findings have been inconclusive regarding the effectiveness of alignment and have generated controversial outcomes regarding pain perception. No significant differences were observed in root resorption between different archwires. However, one study indicated the potential benefits of monitoring root resorption using Superelastic-NiTi wires. 19

A Cochrane review of aligning archwires performed by Wang et al 1 examined various aligning archwires and found inadequate evidence to decide which archwire material was better regarding the alignment efficiency, associated perception of pain, and the possibility of inducing root resorption. The study also highlighted the necessity to perform additional randomized clinical trials (RCTs) to ascertain the optimal initial wire.

Only a few clinical trials have been designed to evaluate the efficiency of newer aligning archwires. Thus, comparing the traditional aligning archwire, such as Superelastic-NiTi, with the more advanced ones becomes necessary. For this reason, this study combined the results of two RCTs (with similar features) 6 21 to compare the effectiveness of Superelastic-NiTi, SmartArch, Cu-NiTi, and Tubular coaxial-NiTi regarding the alignment efficiency, associated perception of pain, and the possibility of orthodontically induced inflammatory root resorption (OIIRR). Each study initially hypothesized that “there was no significant difference in the effectiveness of the compared aligning archwires during the early phase of orthodontic therapy.”

## Aim and Objectives

### Aim

The study aimed to assess the effectiveness of four aligning archwires in the initial stage of treatment.

### Objectives

#### Primary Objective

To compare the degree of alignment of lower anterior teeth obtained at 4, 8, 12, and 16 weeks from the bonding day among the study groups.

#### Secondary Objectives

- To compare the degree of the patient's perception of pain throughout the initial 7 days after each archwire insertion among study groups.
- To compare the amount of OIIRR that takes place in the root apex of the mandibular central incisors among the study groups.

## Materials and Methods

### Trial Design and Any Changes after Trial Commencement

This study combined the results of two RCTs, 6 21 both of which were designed to be a multicenter prospective blinded RCT with parallel groups and an equal allocation ratio. A randomization without stratification was performed. Registration of both trials in ClinicalTrials.gov was done, one on May 26, 2022 (Registration number: NCT05391542) and the other on August 18, 2022 (Registration number: NCT05510206). There were no changes to the methodology after the trial commencement.

### Participants, Eligibility Criteria, and Setting

The trials were performed in different centers: governmental centers specialized in orthodontics, Department of Orthodontics/College of Dentistry/University of Baghdad, and private orthodontic clinic in Baghdad/Al-karkh district by specialist orthodontists or orthodontists under training. Participation in the trials was provided to patients who fulfilled the eligibility criteria and visited these centers for orthodontic treatment using fixed appliances. The same inclusion and exclusion criteria were applied in both trials as follows.

#### Inclusion Criteria

- Patients aged approximately 12 years or older with mandibular anterior teeth crowding of 5 to 9 mm, as determined by Little's irregularity index (LII), who are indicated for fixed appliance orthodontic therapy without extraction.
- With the exception of the third molars, all permanent lower teeth have erupted.
- The overbite and the overjet should not impede the bonding of brackets on the lower anterior teeth.
- No previous history of root resorption or trauma in the region of mandibular incisors.

#### Exclusion Criteria

- Previous orthodontic treatment using fixed appliance.
- Mandibular incisor crowding of less than 5 mm (LII).
- Cases with severe crowding (LII > 9 mm) or cases with blocked-out teeth that make the engagement of the aligning archwires impossible or necessitate extraction of teeth in the mandibular arch.
- A history of periodontitis and attachment loss.

At first, the investigators assessed the patients' eligibility according to the predetermined inclusion criteria. The patients who satisfied the inclusion criteria received adequate information about the study written in a patient information sheet to thoroughly read at home before deciding whether or not to participate. The participants were then requested to sign the consent forms. For patients under the age of 18 years, parental consent was obtained.

### Interventions

The treatment protocol was standardized in both trials, and no changes to the protocol were allowed. The following bonding procedure was used: teeth polishing, rinsing and drying, etching using 37% phosphoric acid, thorough rinsing and drying, and bonding the brackets (Pinnacle, MBT prescription, Ortho Technology, West Columbia, South Carolina, United States) and molar tubes (Buccal tubes, Ortho Technology, West Columbia, South Carolina, United States) using a height gauge (Bracket Height Gauge, Ortho Technology, West Columbia, South Carolina, United States) and light-cured adhesive (Light Bond, Reliance, Itasca, United States). For each group, the archwires were placed in the following sequence:

1. Superelastic-NiTi (TruFlex Nickel-Titanium, Ortho Technology, West Columbia, Illinois, United States):
  - 0.014 inch.
  - 0.018 inch.
2. SmartArch (SmartArch Laser Engineered Copper Ni-Ti, Ormco, California, United States):
  - 0.016 inch
3. Cu-NiTi (Damon Optimal-Force Copper Ni-Ti, Ormco, California, United States):
  - 0.014 inch.
  - 0.018 inch.
4. Tubular coaxial-NiTi (Speed tubular supercable, Speed System Orthodontics, Ontario, Canada):
  - 0.016 inch.
  - 0.018 inch.

On the bonding day, the first archwire in the sequence was placed for all groups. Eight weeks later, the first archwire was replaced by the second archwire for another 8 weeks except for the SmartArch group, in which the same size archwire (0.016 inch) remained for the whole 16 weeks. Elastic modules (Ortho Technology, West Columbia, South Carolina, United States) were used to ligate archwires to all mandibular teeth brackets, and the modules were ligated in the same way for all participants. To prevent any bias to the results other than the variation in archwire material, no stoppers or bendbacks were used in all groups. Patients were requested to report any debonding incident. If a debond was reported, the case was handled as an emergency and rebonded within 24 hours. However, failure to report debond results in the patient being considered dropped out. Patients were reminded through phone calls 3 to 4 days before their scheduled appointments.

Utilizing an alginate impression material (Hydrogum, Zhermack, Badia Polesine, Italy), a high-quality impression for the mandibular arch was made prior to treatment and then every 4 weeks for the next 16 weeks: at 4, 8, 12, and 16 weeks. Each impression was then cast using a type IV dental stone (Elite Stone, Zhermack, Badia Polesine, Italy). For each participant, five study casts were thus produced. A Visual analogue scale (VAS) was provided to the participants so they could mark the pain level felt throughout the initial 7 days after each archwire insertion. Using the long cone paralleling technique, every participant had two periapical radiographs for their mandibular central incisors, one pretreatment and the other at 16 weeks.

### Outcomes and any Changes after Trial Commencement

#### Primary Outcome (Effectiveness of Alignment)

The LII was used to measure the alignment of the six anterior mandibular teeth. 19 The linear displacement of five anatomic contact points between the mesial contact point of the right mandibular canine and the left mandibular canine was calculated to the nearest 0.01 mm. 22 An electronic Vernier caliper (Aluminum Caliper 4” Digital caliper, IOS, Stafford, United States) was used to measure LII directly on defect-free mandibular study casts.

### Secondary Outcomes

#### Pain Perception

A 10-cm long line with a 10-point VAS was used to measure the patient's pain level. During the first 7 days after each archwire insertion, a sheet with a VAS (one for each of the 7 days) was handed to each patient, and they were told to mark a score that represented the highest level of pain they experienced each day at night (0 represented no pain and 10 represented unbearable pain). Patients were advised to document the level of pain they felt in the mandibular teeth before taking any analgesics. 19

#### Root Resorption

The evaluation of root resorption was performed pretreatment and after 16 weeks. A portable X-ray machine (Hyperlight Portable X-ray Unit, Eighteeth, Jiangsu, China) and sensor (Nanopix 2, Eighteeth, Jiangsu, China) were used to take periapical radiographs for the mandibular central incisors. The settings for the portable X-ray machine were 2 mA DC, 70 kV, and 0.16 seconds exposure time. The X-ray tube was placed using a sensor holder at a fixed distance of 7 cm from the sensor, applying a standardized long-cone paralleling technique. The root resorption was assessed using the Malmgren et al's 23 scoring system. The highest score for the right or left lower central incisors was selected. 24 There are five grades in this scoring index:

- *Grade 0:* Apical root resorption is absent.
- *Grade 1:* Irregular contour of the root apex.
- *Grade 2:* Minor resorption of the root apex less than 2 mm.
- *Grade 3:* Severe resorption of the root apex of 2 mm—1/3 of the original root length.
- *Grade 4:* Extreme resorption of the root apex of more than 1/3 of the original root length.

There were no outcome changes after trial commencement.

### Sample Size Calculation

Based on an RCT by Nabbat and Yassir, 19 the sample size for the two trials was calculated with a power of 80% and a 5% α level to identify a 1.5-mm difference in the degree of crowding alleviation between groups. This showed that 13 participants in each study arm would be needed to detect this clinical difference. To account for a dropout, a total of 73 patients were recruited.

### Randomization

#### Sequence Generation

Using a computer random generator, simple randomization with no stratification and a 1:1 allocation ratio was produced for each trial. The patient ID was represented by the sequential numbers in the generated random table based on the trial's enrollment priority. An independent person randomly coded the allocation groups, and the coding remained concealed from the investigators until the measurement and analysis of the data were finished.

#### Allocation Concealment

Allocation concealment was accomplished by using sealed opaque envelopes numbered consecutively according to the patient's ID. Three items were inserted into each envelope: a card bearing the allocation group number, brackets set and elastomeric modules, and related archwires numbered in order of use. The envelope remained closed, and the clinician was unable to know the allocation group for each patient until the day of bonding.

### Blinding

Since the trials were performed in multiple clinics, the ID numbers used to identify participants were written on all the trial documents. This allowed for the completion of data collection and measurements while keeping both the investigators and the data analyst blinded to the allocation groups. However, blinding the clinicians during archwire insertion was not possible as the clinicians could recognize the types of archwires based on their flexibility and appearance.

### Stopping Guidelines

It was decided to stop the trials if any group experienced severe pain that the participants could not tolerate.

### Statistical Analysis

Data were analyzed using the Statistical Package for the Social Sciences for Windows (SPSS Inc., Version 25.0, Chicago, Illinois, United States). The subsequent statistical analyses were conducted.

#### Descriptive Statistics

Numbers, frequencies, percentages, minimum, maximum, mean, median, standard deviation, and interquartile range were included.

#### Reliability Statistics

- *LII* : 20 study models were assessed for inter- and intra-examiner reliability using an intraclass correlation coefficient (ICC) test. Two measurements were performed at an interval of 4 weeks.
- *Root resorption* : 20 periapical radiographs were assessed for inter- and intra-examiner reliability using the weighted kappa test. Two separate scoring were made at an interval of 4 weeks.

**Note:** Inter-examiner reliability for the two trials was performed by two investigators and two experts, who measured 20 study models and scored 20 periapical radiographs.

#### Inferential Statistics

The normality of the data distribution of the dependent variables was examined using the Shapiro–Wilk test. On the other hand, Levene's test was employed to verify the group's homogeneity. The following analyses were performed:

- *Repeated measures analysis of variance (ANOVA) test with mixed factorial design (between and within-subject effect):* to determine whether there were differences between the four groups in the amount of crowding relief with time interaction.
- *Kruskal–Wallis test (with pairwise comparison):* to determine if there was a difference between the four groups in the perception of pain and root resorption.
- *Wilcoxon signed-rank test:* to compare the amount of root resorption pretreatment and after 16 weeks within each group.

A significance level of *p* <0.05 was applied.

## Results

### Participant Flow

**Fig. 1:**
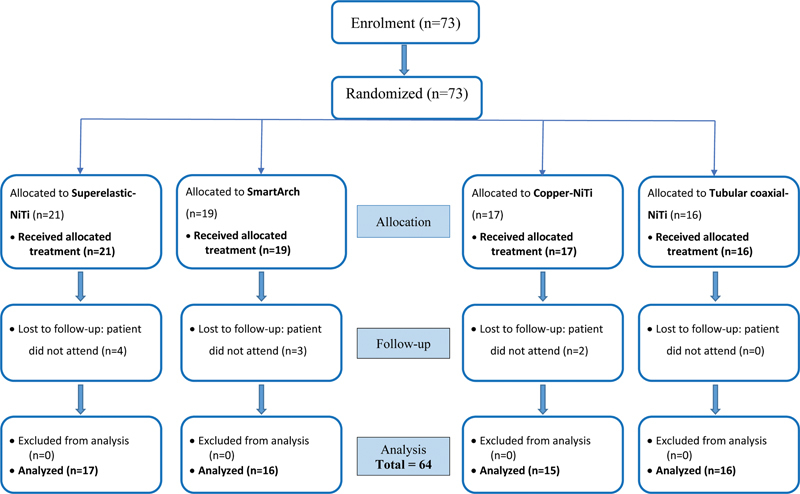
The CONSORT flowchart of the participants for the clinical trials.

Patients were recruited from February 2022 until August 2022 fulfilling the required sample size for each trial. Seventy-three patients were enrolled in both trials (21 were allocated randomly to the Superelastic-NiTi, 19 to the SmartArch, 17 to the Cu-NiTi, and 16 to the Tubular coaxial-NiTi groups). Before the end of the trials, nine patients dropped out as they either failed to show up for the scheduled appointments, underwent debonding that could not be rebonded within 24 hours, or removed the appliance. The analysis included the 64 patients who completed the trials (17 in Superelastic-NiTi, 16 in SmartArch, 15 in Cu-NiTi, and 16 in Tubular coaxial-NiTi groups). The Consolidated Standards of Reporting Trials (CONSORT) flowchart of the study participants is shown in Fig. 1 .

### Baseline Data

The pretreatment baseline data of the participants are shown in Table 1 . The results did not show any statistically significant difference in baseline characteristics between the groups.

**Table 1:** Baseline characteristics of the participants in each study group

Baseline characteristics		Superelastic-NiTi ( *N* = 17)		SmartArch ( *N* = 16)		Copper-NiTi ( *N* = 15)		Tubular coaxial-NiTi ( *N* = 16)		*p*
Continuous data		Mean	SD	Mean	SD	Mean	SD	Mean	SD	
**Age**		16	1.87	18	5.4	16	2.1	15	2.16	0.073 a
**Start LII**		6.82	1.39	7.17	1.31	6.57	1.15	7.46	1.4	0.261 a
**Categorical data**		**Count**	**%**	**Count**	**%**	**Count**	**%**	**Count**	**%**	
**Gender**	Female	12	70.6	9	56.3	11	73.3	11	68.8	0.745 b
	Male	5	29.4	7	43.8	4	26.7	5	31.3	
**Pre-treatment root resorption scores**	0	6	35.3	10	62.5	12	80.0	11	68.8	0.049 c
	1	10	58.8	6	37.5	3	20.0	5	31.3	
	2	1	5.9	0	0.0	0	0.0	0	0.0	

Abbreviation: LII, Little's irregularity index; NiTi: Nickel-Titanium.

a ANOVA test.

b Chi-square test.

c Kruskal–Wallis test.

The assumptions of normality of data distribution for the dependent variables and homogeneity of the data between groups (equality of error variances) were not violated, as revealed by the Shapiro–Wilk and Levene's tests ( *p* > 0.05).

### Reliability Tests

The ICC results for inter- and intra-examiner reliability for LII measurements in both trials 6 21 showed excellent agreement (inter-examiner reliabilities were 0.996 and 0.998, respectively; intra-examiner reliabilities were 0.998 and 0.996, respectively).

Weighted kappa test also revealed adequate inter- and intra-examiner reliability for root resorption measurements in both trials (inter-examiner reliabilities were 0.8 and 0.783, respectively; intra-examiner reliabilities were 0.8 and 1.00, respectively).

### Numbers Analyzed for Each Outcome, Estimation, Precision, and Subgroup Analyses

A total of 73 patients were enrolled in both trials, nine of whom, making up 12.3% of the total sample, had been dropped out (within the accounted number of dropouts). The analyzed sample attained a power of greater than 80%. Since the participants of both trials were collected according to the same eligibility criteria, there were no qualitative differences among them.

Table 2 shows the descriptive data for treatment outcomes at different time points during the trials. The four groups started with slightly different means for LII (6.82 mm for Superelastic-NiTi, 7.17 mm for SmartArch, 6.57 mm for Cu-NiTi, and 7.46 mm for Tubular coaxial-NiTi). However, their final means at week 16 were comparable.

**Table 2:** The descriptive statistics for treatment outcomes (teeth irregularity, pain, and root resorption) at different time points

Variable		Superelastic-NiTi		SmartArch		Copper-NiTi ( *N* = 15)		Tubular coaxial-NiTi ( *N* = 16)	
**LII**	**Time**	**Mean**	**SD**	**Mean**	**SD**	**Mean**	**SD**	**Mean**	**SD**
	Pretreatment	6.82	1.4	7.17	1.31	6.57	1.15	7.46	1.4
	4 weeks	4.65	1.51	3.98	1.50	3.40	1.09	4.73	2.04
	8 weeks	2.84	1.02	2.84	1.36	2.34	1.08	2.77	1.59
	12 weeks	2.19	0.77	2.11	1.13	1.78	0.93	1.84	1.17
	16 weeks	1.53	0.72	1.54	1.18	1.35	0.75	1.43	0.91
	8-week change	−3.98	1.17	−4.33	1.62	−4.23	1.56	−4.69	1.64
	16-week change	−5.29	1.47	−5.63	1.55	−5.22	1.44	−6.03	1.45
**Pain perception**	**Day**	**Median**	**Interquartile range**	**Median**	**Interquartile range**	**Median**	**Interquartile range**	**Median**	**Interquartile range**
**1st wire**	1st	5	6	6	6	6	6	6	5
	2nd	5	6	4.5	5	3	7	5	3
	3rd	3	5	2	4	2	5	1.5	5
	4th	1	4	2	4	1	3	1	3
	5th	0	4	2	3	1	3	0	2
	6th	0	0	0	3	0	1	0	1
	7th	0	0	0	2	0	1	0	0
**2nd wire**	1st	5	6			3	4	1.5	6
	2nd	0	5			2	4	0	5
	3rd	0	3			1	3	0	3
	4th	0	0			0	1	0	2
	5th	0	0			0	1	0	0
	6th	0	0			0	0	0	0
	7th	0	0			0	0	0	0
**Root resorption**	**Score**	**Count**	**%**	**Count**	**%**	**Count**	**%**	**Count**	**%**
**Pretreatment**	0	6	35.3	10	62.5	12	80.0	11	68.8
	1	10	58.8	6	37.5	3	20.0	5	31.3
	2	1	5.9	0	0.0	0	0.0	0	0.0
**16 weeks of treatment**	0	0	0.0	3	18.8	5	33.3	5	31.3
	1	10	58.8	9	56.3	8	53.3	10	62.5
	2	7	41.2	4	25.0	2	13.3	1	6.3

Abbreviations: NiTi, Nickel-Titanium; LII, Little's irregularity index; SD, standard deviation.

Note: 8- and 16-week LII changes were considered from the pretreatment scores.

**Fig. 2:**
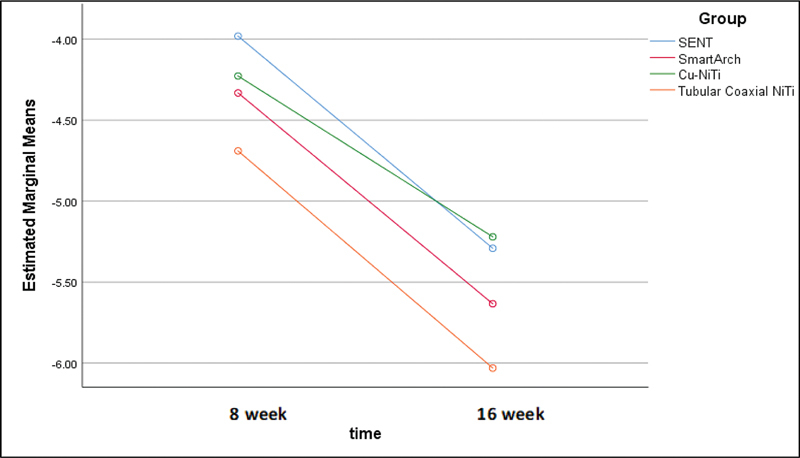
Profile plots for the change in LII over time for each group. LII, Little's irregularity index.

According to the mixed factorial ANOVA test, the change in LII with time was statistically significant for all groups ( *p* = 0.000). However, the same test showed no statistically significant differences between the four groups ( *p* = 0.482); similarly, the time*group interactions for all groups were not significant ( *p* = 0.623; Table 3 ). At the end of the trials, the greatest change in LII (although statistically nonsignificant) was shown in the Tubular coaxial-NiTi group, followed by the SmartArch group according to the profile plots ( Fig. 2 ).

**Table 3:** Repeated measure mixed factorial design ANOVA test to compare study groups based on how Little's irregularity index changed with the time factor

Source	Type III sum of square	df	Mean square	*F*	*p*
**Time**	48.83	1	48.83	142.14	**0.000**
Time * Group	0.61	3	0.20	0.59	0.623
**Group**	10.22	3	3.41	0.83	0.482

Abbreviation: ANOVA, analysis of variance.

Note: Sphericity assumed. *p* -Value in bold indicates statistical significance.

The first archwire caused higher pain perception than the second archwire in the three groups (Superelastic-NiTi, Cu-NiTi, and Tubular coaxial-NiTi; Table 2 ). The Kruskal–Wallis test revealed nonsignificant differences between the four groups regarding the perception of pain with the first archwire except for the seventh day ( *p* = 0.008), in which the pain was statistically significantly higher in the SmartArch group when compared with the Superelastic-NiTi group ( *p* = 0.009; Tables 4 and 5 ). The Kruskal–Wallis test was also used to compare the perception of pain with the second archwire between the three groups (Superelastic-NiTi, Cu-NiTi, Tubular coaxial-NiTi), and the results revealed nonsignificant differences between them for the 7 days after the insertion of the second archwire *p* > 0.05; Table 6 ).

**Table 4:** Kruskal–Wallis test for comparison of pain perception for the 1st wire used in the four groups

Pain perception		Superelastic-NiTi ( *N* = 17)	SmartArch ( *N* = 16)	Copper-NiTi ( *N* = 15)	Tubular coaxial-NiTi ( *N* = 16)	*p*
Archwire	Day	Mean rank	Mean rank	Mean rank	Mean rank	
** 1 ^st^**	**1st**	25.18	32.66	34.47	38.28	0.222
	**2nd**	32.91	32.47	32.60	32.00	0.999
	**3rd**	37.65	31.88	29.43	30.53	0.579
	**4th**	30.53	35.59	31.20	32.72	0.857
	**5th**	31.47	36.22	33.43	29.00	0.679
	**6th**	26.79	37.97	34.37	31.34	0.179
	**7th**	25.50	40.41	35.60	29.13	**0.008**

Abbreviation: NiTi, Nickel-Titanium.

Note: *p* -Value in bold indicates statistical significance.

**Table 5:** Pairwise comparison of pain perception difference between groups in the 7th day of the 1st wire

Groups	*p*
**Superelastic-NiTi-Tubular coaxial-NiTi**	1.000
**Superelastic-NiTi- Copper-NiTi**	0.204
**Superelastic-NiTi-SmartArch**	**0.009**
**Tubular coaxial-NiTi-Copper-NiTi**	1.000
**Tubular coaxial-NiTi- SmartArch**	0.106
**Copper-NiTi-SmartArch**	1.000

Abbreviation: NiTi, Nickel-Titanium.

Note: *p* -Value in bold indicates statistical significance.

**Table 6:** Kruskal–Wallis test for comparison of pain perception for the 2nd wire used in three groups

Pain perception		Superelastic-NiTi ( *N* = 17)	Copper-NiTi ( *N* = 15)	Tubular coaxial-NiTi ( *N* = 16)	*p*
Archwire	Day	Mean rank	Mean rank	Mean rank	
**2nd**	**1st**	25.03	27.37	21.25	0.453
	**2nd**	23.91	25.90	23.81	0.882
	**3rd**	23.62	27.33	22.78	0.552
	**4th**	22.38	26.10	25.25	0.613
	**5th**	23.00	27.33	23.44	0.377
	**6th**	22.79	24.53	26.28	0.461
	**7th**	23.00	24.53	26.06	0.326

Abbreviation: NiTi, Nickel-Titanium.

The distribution of root resorption scores in each group pretreatment and after 16 weeks is illustrated in Table 2 . Pretreatment scores included only scores 0 and 1 in the SmartArch, Cu-NiTi, and Tubular coaxial-NiTi groups, while in the Superelastic-NiTi group, score 2 was also recorded in addition to scores 0 and 1 in a percentage of 5.9%. Post-alignment scores included scores 0, 1, and 2 in different percentages, and these scores were worse than the pretreatment scores for all groups. The Kruskal–Wallis test showed statistically significant differences between the groups regarding the post-alignment root resorption ( *p* = 0.014), and the pairwise comparison revealed that the difference was between the Superelastic-NiTi and Tubular coaxial-NiTi ( *p* = 0.022), as the former group showed a higher root resorption ( Tables 7 and 8 ). The Wilcoxon signed-rank test revealed a statistically significant root resorption after 16 weeks within each group ( *p* < 0.05; Table 9 ).

**Table 7:** Kruskal-Wallis test for the difference between study groups in post-alignment root resorption

X-Ray	Group	N	Mean Rank	*P*
**16 weeks**	**Superelastic-NiTi**	17	42.50	**0.014**
	**SmartArch**	16	33.69	
	**Copper-NiTi**	15	27.07	
	**Tubular coaxial-NiTi**	16	25.78	

Abbreviation: NiTi, Nickel-Titanium.

**Table 8:** Pairwise comparison of post-alignment root resorption difference between groups

Groups	*p*
Tubular coaxial-NiTi-Copper-NiTi	1.000
Tubular coaxial-NiTi- SmartArch	1.000
Tubular coaxial-NiTi-Superelastic-NiTi	0.022
Copper-NiTi- SmartArch	1.000
Copper-NiTi- Superelastic-NiTi	0.050
SmartArch-Superelastic-NiTi	0.755

Abbreviation: NiTi, Nickel-Titanium.

**Table 9:** Wilcoxon signed-rank test for the difference in root resorption within each group (pretreatment and after 16 weeks)

Group	16-week Pretreatment	*N*	Mean rank	Sum of ranks	*p*
**Superelastic-NiTi**	Negative ranks	0 a	0.00	0.00	**0.001**
	Positive ranks	12 b	6.50	78.00	
	Ties	5 c			
	Total	17			
**SmartArch**	Negative ranks	0 a	0.00	0.00	**0.002**
	Positive ranks	10 b	5.50	55.00	
	Ties	6 c			
	Total	16			
**Copper-NiTi**	Negative ranks	0 a	0.00	0.00	**0.003**
	Positive ranks	9 b	5.00	45.00	
	Ties	6 c			
	Total	15			
**Tubular coaxial-NiTi**	Negative ranks	0 a	0.00	0.00	**0.008**
	Positive ranks	7 b	4.00	28.00	
	Ties	9 c			
	Total	16			

Abbreviation: NiTi, Nickel-Titanium.

Note: *p* -Values in bold indicate statistical significance.

a 16 weeks < pretreatment.

b 16 weeks > pretreatment.

c 16 weeks = pretreatment.

### Harms

No harms were reported during the treatment except for a slight discomfort and root resorption, which are common side effects of treatment with fixed orthodontic appliances.

## Discussion

### Study Design and Sample Characteristics

This study combined the results of two trials with the same inclusion and exclusion criteria to compare the clinical performance of four aligning archwires (Superelastic-NiTi, SmartArch, Cu-NiTi, and Tubular coaxial-NiTi). Apart from for the slight differences in pain perception between the SmartArch and Superelastic-NiTi groups, and the slight differences in root resorption between the Tubular coaxial-NiTi and Superelastic-NiTi, the evidence was insufficient to reject the null hypothesis for the alignment efficiency, but it could be partially rejected for the pain perception and OIIRR.

The recruiting age was set at approximately 12 years and older to ensure that all the permanent teeth are present and root formation has been completed for the mandibular anterior teeth. It was also set to enhance the generalizability by involving both adolescent and adult groups.

All groups showed a higher female-to-male ratio, which is in line with other studies. 19 25 26 This may be related to the higher esthetic demand of the females as compared with males.

### Intervention

In this study, the archwire sequence used in the Superelastic-NiTi and Cu-NiTi groups was 0.014 inch followed by 0.018 inch, which is in accordance with one study that used the same sequence when comparing the same archwires, 7 but partially agrees with two studies that used 0.014-inch archwires first, followed by 0.016 inch. 25 27

However, the Tubular coaxial-NiTi and SmartArch groups started with 0.016 inch. The 0.016 inch is the smallest diameter for Tubular coaxial-NiTi archwire produced by the Speed System Orthodontics, and based on the comparison chart of the unloading forces available from the manufacturer ( www.speedsystem.com ), this size exerts roughly a quarter of the force of the 0.013-inch Cu-NiTi wire. This comes in line with one study that used the same diameter of archwire in their study. 12 For the SmartArch, the size 0.016 inch was used for the whole 16 weeks to comply with the manufacturer's advice, which states that this size should be kept until alignment is obtained, and then a 0.018 × 0.025-inch SmartArch wire should be used next.

The amount of crowding selected in this study (5–9 mm LII) as well as the anatomy of the mandibular anterior teeth (with shorter inter-bracket distance) could both enhance the superelastic behavior of the first archwires used in this study, since the least amount of archwire deflection needed to express this behavior is 2 mm. 12 28 29

In this study, an interval of 8 weeks was applied for changing archwires. This is in line with various studies 7 30 31 and also comes in accordance with the type of archwires that require long-term use as recommended by the manufacturers.

### Alignment Efficiency

The results of this study showed that there was a statistically insignificant difference in the alignment efficiency among the four archwires. This finding agreed with other studies in which NiTi archwires, in their various forms such as Superelastic-NiTi, Heat-activated-NiTi, and Cu-NiTi, were found to be equally effective in relieving crowding. 3 4 7 19 25 32 In contrast, one study found that the Heat-activated-NiTi archwires performed better than the Superelastic-NiTi in relieving crowding. 33 The results of the current study also agreed with another study that found no significant difference between single-strand and multi-strand NiTi 12 and disagreed with two studies that found significant differences favoring the coaxial NiTi archwires over single-stranded NiTi archwires. 10 34

When interpreting the results closely, the Tubular coaxial-NiTi followed by the SmartArch may have acted slightly better than the other archwires as both started with higher LII scores but ended with a comparable LII with the other groups. This may be due to the greater degree of rotational control offered by the Tubular coaxial-NiTi, as it engages easily into brackets even in severe malalignment cases. For the SmartArch, the reason could be attributed to the optimum force applied to each tooth, which can lead to more efficient tooth movement and a reduction in treatment time. However, this difference was deemed statistically nonsignificant.

### Pain Perception

Pain and discomfort caused by orthodontic treatment should be considered seriously in orthodontics since they play a major role in denying or discontinuing the treatment. 35 36 37 Thus using an archwire that causes lower pain perception is of high clinical importance.

VAS was used in this study to evaluate pain perceived by the patients because it is simple and reliable. It has also been successfully applied in other trials for evaluating the pain perception among different aligning archwires. 18 19 26 27 38

After bonding, pain and discomfort were assessed every night for 7 days following the insertion of each aligning archwire, while some studies kept the VAS data open for a month after the insertion of archwires. 27 Discomfort is often felt in the first week following bonding, as the biological reaction to orthodontic stresses is reflected in the pain-time pattern. The concentration of interleukin-1β in gingival crevicular fluid, which causes the production of molecules that cause pain, rises after an hour, peaks after 24 hours, and then levels out in a week or a month. 39 So, it is clinically relevant to identify the difference in pain level within this interval.

SmartArch and Tubular coaxial-NiTi were thought to cause a lesser amount of pain perceived by the patient since the first one was supposed to deliver optimum forces for different teeth and the second one was supposed to deliver lighter forces than the Superelastic-NiTi and Cu-NiTi. However, this study results showed no statistically/clinically significant differences between the four groups regarding the first archwire except for a slightly significant difference between the SmartArch and Superelastic-NiTi on the seventh day favoring the latter. The nonsignificant difference between the archwires may be due to the individual variation in the perception of pain among patients. This result came in line with various other studies that compared different types of aligning archwires, 3 4 19 38 while disagreeing with another study that compared Heat-activated-NiTi and Superelastic-NiTi and found a significant difference favoring the Heat-activated-NiTi archwires. 18

The result of the comparison of the second archwire in the Superelastic-NiTi, Cu-NiTi, and Tubular coaxial-NiTi groups showed no statistically significant differences. These results showed lesser pain perception as compared with the first archwires, which is anticipated because the highest pain perception is perceived at the initial archwires after the first activation of the fixed orthodontic appliance. 30 40

### Root Resorption

It was challenging to compare the findings of this study to earlier studies since other RCTs differ from this one in terms of the teeth that were selected for assessment, the type of radiograph utilized, the technique and timing of the evaluation, or the archwire sequence. There was a statistically significant difference between the Superelastic-NiTi and Tubular coaxial-NiTi groups. The Tubular coaxial-NiTi archwires exert only a fraction of the force exerted by the traditional Superelastic-NiTi archwires, and this could be the reason for the lower post-alignment scores of the Tubular coaxial-NiTi group. Otherwise, no statistical significance was found when comparing other groups in OIIRR, and this came in agreement with other studies that reported the amount of OIIRR was not affected by the kind or sequencing of the archwires. 18 19 41 Likewise, this result came in line with an overview of systematic reviews regarding OIIRR, which concluded that varied archwire type and sequences had the same OIIRR. 42

The degree of root resorption at baseline and after 16 weeks was compared within each group, and there were significant differences in the four groups. This might be attributed to the continuous force applied by the four wires, 43 which may prevent the resorbed cementum from healing. 19 42 44 However, it did not seem that the examined archwires resulted in unacceptably high levels of iatrogenic root resorption, as the clinically significant root loss is defined to be more than 2.5 mm. 41 Almost all orthodontic patients undergo some resorption, according to previous reports, 45 and radiographic examinations of orthodontic patients who had extensive treatment showed some root resorption in most teeth. 30 It often manifests mildly with a less than 2.5 mm radiographic mean. 46

### Strengths and Limitations of the Study

#### Strengths

Four different types of aligning archwires have been compared in this study, including two advanced types.

The included trials complied with the CONSORT group principles and were immediately registered with ClinicalTrials.gov following protocol review and ethical approval.

Both trials were conducted in different centers (general and private) and included both adolescent and adult participants, which could enhance the generalizability of the outcomes. In addition, the multicenter RCT design is the ideal design for assessing the clinical efficacy of aligning archwires according to the guidelines given by the systematic reviews about the aligning archwires. 1 47

Sufficient randomization and allocation concealment were implemented to minimize the bias, which was reflected by the nonsignificant differences in the baseline characteristics among groups.

Both trials followed the same treatment protocol, which could enhance the reliability of the findings.

#### Limitations

The variations in the arch form of the included archwires could potentially affect the results. Even so, the extent of the expansion was outside of the scope of this study, and the wires are not rigid enough to affect arch width.

Since there was only one size of round SmartArch wire, pain perception caused by this wire was compared with other wires for the initial wire only. Furthermore, characteristics such as age, sex, and psychological aspects like depression, anxiety, and changes in female hormones during menstruation were not taken into account when evaluating pain perception.

Another shortcoming of the current research is the short period that root resorption was reported; however, a prolonged follow-up period would extend the course of treatment, which might not be ethically justified.

Although the power of both included trials exceeds 80%, the potential advantage of Tubular coaxial-NiTi and SmartArch wires could be noticeable if the sample size was increased.

For future development of this line of research and to address some of the above limitations, the authors suggest increasing the sample size, conducting the same trial on patients with severe crowding who need extraction, as this might demonstrate a difference in the clinical performance of these wires, improving the methodology by using three-dimensional (3D) imaging technology to evaluate OIIRR in three dimensions, using intraoral scanner to obtain 3D study models and using various types of software to measure the LII, and addressing the confounding effect of the factors that affect pain perception. Additionally, other variables such as flexural strength, 48 cytotoxicity, 49 and metal release 50 should be considered in future studies.

### Clinical Implications

The results of this study showed that using the newer and more costly archwires is similar in correcting malalignment of the mandibular anterior teeth when compared to the traditional archwires that are already available in the market.

Moreover, this study supports the recommendation that all patients should be monitored radiographically for root resorption, starting in the early stages of orthodontic treatment.

## Conclusion

- Superelastic-NiTi, SmartArch, Cu-NiTi, and Tubular coaxial-NiTi archwires performed comparably well during the alignment stage of treatment.
- Pain perception was comparable with the four studied archwires.
- There was evidence of some root resorption, which may need observation when using any of these archwires, but there was no significant difference among them.
- As this study did not find any therapeutic benefits of one archwire over another, so orthodontist preference and cost of the wire could be considered when choosing an initial archwire.
